# Rhinitis, sinusitis and ocular disease – 2097. Dentomaxilofacial disorders in children with allergic rhinitis

**DOI:** 10.1186/1939-4551-6-S1-P173

**Published:** 2013-04-23

**Authors:** Claudia Ivonne Gallego, Sandra Nora Gonzalez, Alfredo Arias, Marisela Hernandez, Roberto Carrillo, Hilda HH Torre, Alejandra Venegas

**Affiliations:** 1 Regional Center of Allergy and Clinical Immunology, University Hospital, Monterrey, Mexico; 2 Allergy and Clinical Immunology, University Hospital, Monterrey, Mexico; 3 Orthodontic, Faculty of Dentistry, Uanl, Monterrey, Mexico

## Background

Allergic rhinitis is the most common chronic rhinitis and is a major cause of mouth breathing in children. There are few studies on the prevalence of tooth and jaw abnormalities in children with allergic rhinitis.

## Methods

Observational, Cross-sectional, comparison study. Approved by University Ethics Committee and were obtained written informed consent by parents and children. We included children from 8 to 14 y, diagnosed with allergic rhinitis with and without asthma. They were evaluated by clinician in Allergy and Clinical Immunology and by expert in Orthodontics. The prevalence of dentomaxilofacial alterations was determinate and the results were compared with a control group. We used Mann-Whitney, Chi-square or Fisher exact statistical test. In addition, there was a risk analysis (OR) for rhinitis. P value <0.005 was statistically significance.

## Results

We studied 48 children: 28 in the allergic rhinitis group and 20 in the control group. The age and gender distribution was homogeneous in both groups (p = 0.28). A half of children with allergic rhinitis had asthma-comorbidity. The 85% (n = 41) of the children had never been consulted by a dentist. In comparison with control group the patients with allergic rhinitis had a higher prevalence mouth breathing (71% vs 5%, p = 0.00), compression of the upper jaw (28% vs 0%, p = 0.008), lip incompetence (46% vs 5%, p = 0.002) and snoring (53% vs 5%, p = 0.00). Children with allergic rhinitis were phenotypically characterized by rings (92%, p= 0.03), vertical facial plane growth (25%, p = 0.016), nasal fold (78%, p = 0.00) and retrognathia (17%, p = 0.057). In risk analysis found that mouth breathing increased 47.5 times the risk of allergic rhinitis. Children with persistent rhinitis and asthma had more mouth breading, snoring and jaw compression than children only with rhinitis.

## Conclusions

Children with allergic rhinitis had higher prevalence of facial, tooth and jaw disorders than children without rhinitis. We recommend a multidisciplinary assessment to identify dentomaxilofacial alterations in this high risk groups and provide early treatment.

